# Against Pungent Odors

**Published:** 1893-07

**Authors:** 


### AGAINST PUNGENT ODORS.

Every one does not know that aromatic salts and very strong, pungent odors are injurious to the nerves of smell, and often produce serious, if not incurable, difficulties. It is well understood that certain scents start the action of the secretory glands of the nose and throat and often the eyes fill up with tears. Frequent indulgence in the use of such perfumes will soon overtask the secretory organs and weaken them. Some day the person observes that the hearing is less acute than usual and the sense of smell seems defective. This is, of course, accredited to a cold or some similar cause, and but little is thought of it. After a time the entire head becomes affected, hearing and smell are almost, if not altogether, lacking, and there are throat and lung complications which are likely to end in chronic, if not fatal illness.

It has taken the medical world a great many years to discover that loss of hearing is almost invariably caused by some disease of the throat or nose, or both. But very recent researches in these fields have demonstrated this fact beyond question, and it is now admitted by the most advanced medical men that aside from rupture of the ear drum there is scarcely a symptom of defective hearing which is not traceable directly to the condition of the nose and throat. In view of the new discoveries, ear specialists are finding their occupations gone, save as they make their particular branch an assistant in further investigation.

It is said that the use of smelling salts is one of the most prolific causes of deafness, operating by weakening the olfactory nerves, and through them the auditory system. All strong or pungent odors should be avoided as far as possible, especially those which act upon the secretory processes and as the popular expression goes, "makes the nose run."

